# Strong genetic structure corresponds to small-scale geographic breaks in the Australian alpine grasshopper *Kosciuscola tristis*

**DOI:** 10.1186/s12862-014-0204-1

**Published:** 2014-10-02

**Authors:** Rachel A Slatyer, Michael A Nash, Adam D Miller, Yoshinori Endo, Kate DL Umbers, Ary A Hoffmann

**Affiliations:** Department of Zoology, The University of Melbourne, Parkville, VIC 3010 Australia; Bio21 Molecular Sciences Institute, The University of Melbourne, Parkville, VIC 3010 Australia; Entomology Unit, South Australian Research and Development Institute, Urrbrae, SA 5064 Australia; Department of Genetics, The University of Melbourne, Parkville, VIC 3010 Australia; Wildlife Research Centre, Kyoto University, Sakyo, Kyoto 606-8203 Japan; School of Biological Sciences, University of Wollongong, Wollongong, NSW 2522 Australia; Centre for Evolutionary Biology, University of Western Australia, Crawley, WA 6009 Australia

**Keywords:** Australian alps, Grasshopper, *Kosciuscola tristis*, Phylogeography, Population genetics, Alpine

## Abstract

**Background:**

Mountain landscapes are topographically complex, creating discontinuous ‘islands’ of alpine and sub-alpine habitat with a dynamic history. Changing climatic conditions drive their expansion and contraction, leaving signatures on the genetic structure of their flora and fauna. Australia’s high country covers a small, highly fragmented area. Although the area is thought to have experienced periods of relative continuity during Pleistocene glacial periods, small-scale studies suggest deep lineage divergence across low-elevation gaps. Using both DNA sequence data and microsatellite markers, we tested the hypothesis that genetic partitioning reflects observable geographic structuring across Australia’s mainland high country, in the widespread alpine grasshopper *Kosciuscola tristis* (Sjösted).

**Results:**

We found broadly congruent patterns of regional structure between the DNA sequence and microsatellite datasets, corresponding to strong divergence among isolated mountain regions. Small and isolated mountains in the south of the range were particularly distinct, with well-supported divergence corresponding to climate cycles during the late Pliocene and Pleistocene. We found mixed support, however, for divergence among other mountain regions. Interestingly, within areas of largely contiguous alpine and sub-alpine habitat around Mt Kosciuszko, microsatellite data suggested significant population structure, accompanied by a strong signature of isolation-by-distance.

**Conclusions:**

Consistent patterns of strong lineage divergence among different molecular datasets indicate genetic breaks between populations inhabiting geographically distinct mountain regions. Three primary phylogeographic groups were evident in the highly fragmented Victorian high country, while within-region structure detected with microsatellites may reflect more recent population isolation. Despite the small area of Australia’s alpine and sub-alpine habitats, their low topographic relief and lack of extensive glaciation, divergence among populations was on the same scale as that detected in much more extensive Northern hemisphere mountain systems. The processes driving divergence in the Australian mountains might therefore differ from their Northern hemisphere counterparts.

**Electronic supplementary material:**

The online version of this article (doi:10.1186/s12862-014-0204-1) contains supplementary material, which is available to authorized users.

## Background

Mountain landscapes form a matrix of mountain-top ‘sky islands’ , high-elevation ridges and intervening low-elevation habitat. This creates a discontinuous and fragmented landscape with features such as valleys and river drainages forming potential barriers to gene flow for species restricted to high elevations [1,2]. The scope of these barriers has changed over time. For example, recent warming and consequent upslope distribution shifts have driven fragmentation of previously continuous alpine populations [3]. In contrast, widespread cooling during the Pleistocene glacial periods (0.7-0.01 Ma) is thought to have created corridors of suitable habitat between currently isolated mountain ranges [4,5]. Such large-scale climatic fluctuations have left signatures in the genetic structure of high-elevation taxa worldwide [2,4,6–8].

**Figure 1:**
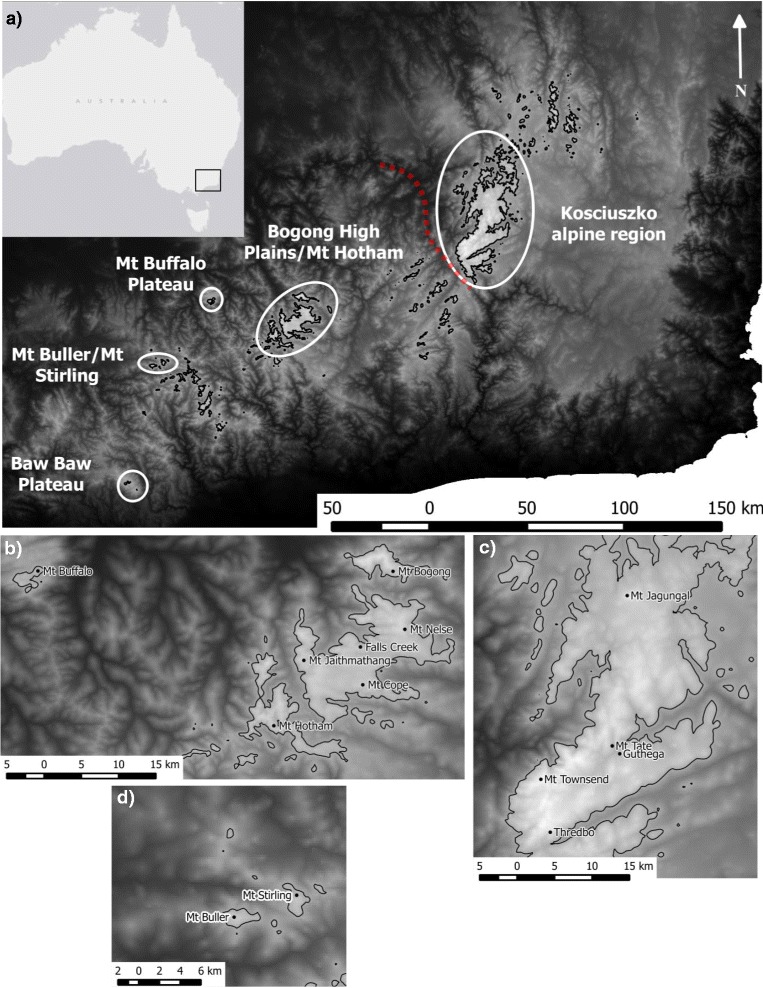
**Map of the Australian alpine area and sampling locations.** The Australian alpine region, showing major mountain areas **(a)** and sampling locations in the Mt Buller/Mt Stirling region **(b)**, Bogong High Plains, Dargo High Plains and Mt Buffalo Plateau **(c)** and the Kosciuszko region **(d)** (see Additional file 1: Table S1 for coordinates). Samples were also collected from the Baw Baw Plateau **(a)**. Black lines indicate a 1500 m contour, the approximate low-elevation distribution limit of *Kosciuscola tristis* through most of its range. Shading represents elevation from 0 m a.s.l (black) to 2250 m (white). The red dotted line in (a) indicates the approximate position of the Murray River, where it divides the Kosciuszko region in New South Wales from the Bogong High Plains area in Victoria. The river also marks the State border, between New South Wales (to the north-east) and Victoria (to the south-west).

During Pleistocene climate cycles, the creation of habitat corridors and tracking of cooler climates to low elevations would have reduced the effective distance between populations and enabled migration of alpine species between previously-isolated mountains [9,10]. Alternatively, glaciation might have driven population contraction to lowland refugia, driving deep lineage divergence across different mountain ranges [2,11–13]. Different patterns are evident across mountain systems that vary in their continuity, extent and ecological characteristics (e.g. dispersal ability) of the focal species. These patterns have most frequently been explored in mountain systems of North America and Europe, which extend across hundreds of kilometres. In Australia, high-elevation areas are highly fragmented but distances between distinct mountain regions are much smaller – usually less than 50 km (Figure 1). Alpine and sub-alpine habitats are confined to a relatively small (5200 km^2^) area of southeastern Australia, with most of this area within the States of Victoria and New South Wales (‘Kosciuszko region’) (Figure 1a) [14]. During the Pleistocene, alpine/sub-alpine conditions would have extended to lower elevations, connecting some, if not all, of these regions [15,16]. At the same time, lack of large-scale glacial activity may have allowed persistence of populations in high-elevation areas [15]. In Victoria (see Figure 1), high endemism and strong divergence among arthropod populations across mountains points to a stable system in which populations have persisted on mountain summits through past climate cycles [17]. Nevertheless, the limited geographic scope of genetic studies in the Australian alps area to-date (see [17,18] for a recent exception) – a consequence of the highly restricted distributions of most Australian alpine taxa – means that patterns of genetic structure across the remainder of the high country remain poorly understood.

Several features of the Victorian and Kosciuszko mountain regions suggest that patterns of historic and contemporary population structure are likely to differ between them. First, while alpine and sub-alpine habitat in Victoria is highly fragmented, currently separated into four main areas by low-elevation agricultural land, the Kosciuszko region comprises largely contiguous alpine/sub-alpine habitat contained within reserved land. Although the Kosciuszko region has few isolated peaks, Mt Jagungal (Figure 1d), which marks the northernmost point of the this region, is separated from the main range by approximately 25 km of sub-alpine plains (~1500 m elevation). Second, the highest areas of the Kosciuszko region experienced some glaciation during Pleistocene climate cycles – the only area of the Australian mainland to do so [19,20]. A recent study of wind-dispersing *Poa* grasses found no genetic structure within the Kosciuszko region, with significant divergence only apparent among isolated mountains in southern Victoria [18]. However, species with low dispersal ability are likely to show greater structure (e.g. [2,11]).

The most extensive areas of alpine and sub-alpine habitat in Australia, lying within the Kosciuszko region in New South Wales and the Bogong High Plains in Victoria, are separated by approximately 100 km of lowland across the Murray River valley, which also marks the State border (Figure 1a). This geographic discontinuity represents a significant divergence point for several alpine reptile and mammalian species [21–23]. For example, the skink *Egernia guthega*, an alpine endemic, shows an average 2.2% mitochondrial (*ND4)* sequence divergence between a population from Kosciuszko and three populations from the Bogong High Plains. However, no genetic structure across this valley was detected among alpine *Poa* [18]. Thus, with the exception of *Poa* [18], the restricted distributions of species studied to-date provides no means to assess the context of the Kosciuszko-Bogong High Plains break within the broader alps area.

In this study, we examine the importance of broad-scale geographic discontinuities in shaping patterns of genetic structure in the Australian high country. In particular, we explore the following questions: (1) at the relatively small spatial scale of Australia’s high country, what is the extent of lineage divergence among and within regions and does the timing of divergence relate to Pleistocene glacial cycles? (2) Are differences in geographic structure and historic climate between the Victorian and Kosciuszko mountains regions reflected in lower historic and contemporary genetic structure in the latter? (3) To what extent does the gap between Kosciuszko and the Bogong High Plains represent a primary divergence point? We investigate these questions using phylogenetic and population genetic frameworks, with markers of different temporal resolutions to capture signatures of both current range fragmentation and potential distribution shifts in the past.

The grasshopper *Kosciuscola tristis* (Sjösted) was identified as an ideal species in which to explore these genetic patterns. Although restricted to Australia’s alpine and sub-alpine habitat, primarily above 1500 m, it is abundant and its distribution stretches from Mt Baw Baw in the south to Mt Jagungal in the north (~300 km). This gives it one of the largest ranges of any mountain-endemic animal in Australia and makes it an ideal model for testing broad-scale phylogeographic patterns in this system. Like many alpine insects, *K. tristis* is flightless and, consequently, is likely to have limited dispersal abilities over the distances separating major mountain regions. Two subspecies are described [24], with *K. tristis tristis* occurring in the Kosciuszko region and *K. tristis restrictus* on the Mt Buffalo plateau (Figure 1). Rehn [24] also describes Victorian populations from Mt Hotham and the Bogong High Plains as morphologically intermediate between the two proposed subspecific forms. We thus predicted (a) genetic divergence across the Murray River valley, and (b) that *K. tristis* from Mt Buffalo would be genetically distinct from other populations of *K. tristis*. Some study has been made of the genetic structure of this species [17,25], providing a basis from which to extend the research to cover a greater geographic extent.

## Results

### Phylogeographic structure and genetic diversity

In total, 75 individuals were sequenced, representing 35 unique *CO1* haplotypes and 12 unique *ITS1* haplotypes. Of these, 12 *CO1* and 3 *ITS1* haplotypes were represented in the Kosciuszko region, with the remainder from populations in Victoria. Mean *CO1* sequence divergence between haplotypes in Victoria was five times that of the Kosciuszko region (Victoria: mean = 2.4 ± 1.1%; Kosciuszko: 0.4 ± 0.2%), and nucleotide diversity was also greater in Victoria (Victoria: $\overline{\pi}$ = 0.006 ± 0.013; Kosciuszko: $\overline{\pi}$ = 0.001 ± 0.001).

**Figure 2:**
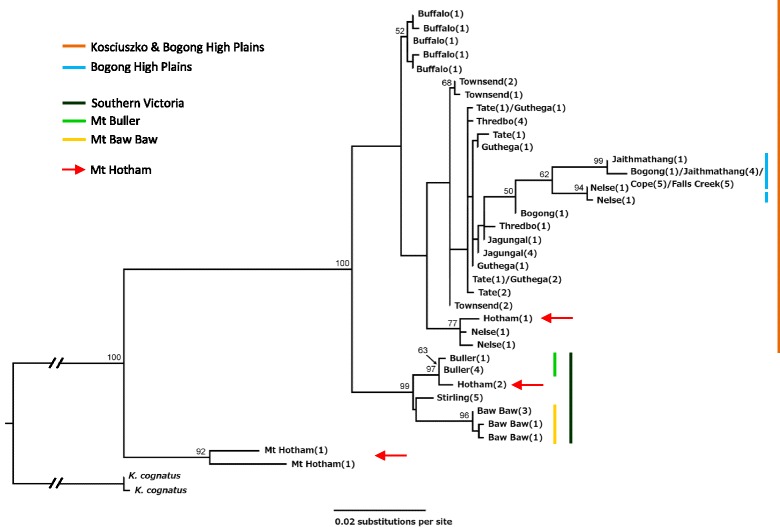
**Maximum likelihood phylogeny of** ***Kosciuscola tristis.*** Maximum likelihood *CO1* phylogeny for *Kosciuscola tristis*, with the congener *K. cognatus* as an outgroup. Populations represented by each haplotype are given in the text next to each tip, with the number of individuals in brackets; coloured bars show phylogeographic clades or (purple bar) geographic affinities, though it should be noted that haplotypes from Mt Hotham (red arrow) occur throughout the phylogeny; bootstrap support values are indicated above the node where support was greater than 50%.

**Figure 3:**
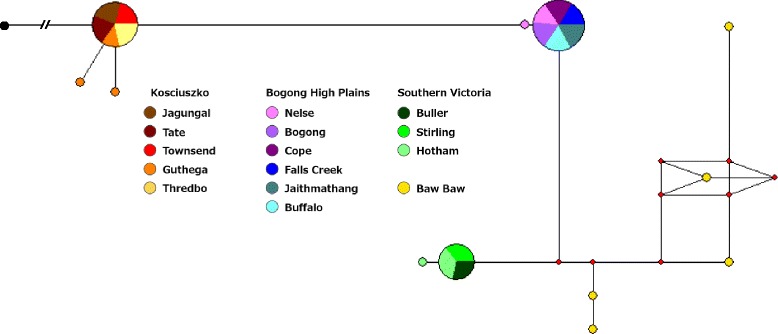
**Median-joining network of** ***Kosciuscola tristis*** **.** Median-joining haplotype network of 12 unique haplotypes from 75 *Kosciuscola tristis ITS1* sequences*,* rooted with *K. cognatus* (black circle)*.* Circles represent haplotypes, with size proportional to haplotype frequencies; for haplotypes common to multiple populations, slices represent the frequencies of each population; colours indicate the population. Branch lengths are proportional to the number of mutations between nodes and red circles are median vectors.

The maximum likelihood *CO1* phylogeny indicates strong statistical support for a southern Victorian clade incorporating populations from Mt Baw Baw, Mt Buller and Mt Stirling. Within this group, further geographic structure was apparent with good support for the monophyly of haplotypes from Mt Baw Baw and from Mt Buller (Figure 2). We found no statistical support for phylogeographic structure across the remaining mountains, although Mt Buffalo (representing the subspecies *K. tristis restrictus*) did cluster. Mt Hotham showed a particularly interesting pattern, with haplotypes falling out in several places on the phylogeny and two individuals – potentially hybrids – showing strong divergence from the other haplotypes. The *ITS1* data, however, placed Mt Hotham with the southern Victorian mountains (Mts Stirling, Buller and Baw Baw). In contrast to the *CO1* phylogeny, *ITS1* sequences showed divergence between the Kosciuszko region and the Bogong High Plains (separated by the Murray River valley), with populations from each region represented by one primary haplotype (Figure 3).

There was an average 2.9% (range 1.9 – 4.1%) sequence divergence between *CO1* haplotypes from the southern Victorian mountains and those from the Bogong High Plains and Kosciuszko. This corresponds to divergence 0.8 to 1.3 Ma (range 0.5 – 1.8 Ma) using a divergence rate of 2.3% or 3.5% Myr^−1^ respectively. Within these southern mountains, divergence of the Mt Baw Baw clade was estimated at 0.4 to 0.7 Ma (range 0.21 – 0.8 Ma) based on a 1.5% (range 0.8 – 1.9%) mean sequence divergence, and the Mt Buller clade at 0.1 Ma (range 0.1 – 0.2 Ma) with 0.3% (range 0.3 – 0.4%) sequence divergence. We were unable to give an estimated divergence time between populations from the Bogong High Plains and Kosciuszko as there was no phylogenetic split based on *CO1* data.

### Microsatellite data: null alleles and diversity

Due to poor amplification of some loci in Victoria (see Methods) we split the data into two. The first dataset consisted of all 13 populations sampled (388 individuals) with five microsatellite loci (five-locus dataset) and the second included the six populations from the Kosciuszko region (177 individuals) and eight loci (Kosciuszko dataset).

The two datasets contained 20% and 14% missing data, respectively. After correcting for multiple comparisons, only one pair of loci at one site showed significant linkage disequilibrium. All loci deviated from Hardy-Weinberg Equilibrium (HWE) in at least one of the sampled sites, with locus *Ktr88* showing the highest level of deviation, departing from HWE at all sites. The other loci that deviated from HWE did so in 4–7 of the seven Victorian populations and in 1–6 of the six Kosciuszko populations. Microchecker [26] indicated the presence of null alleles at four loci: *Ktr30, Ktr58, Ktr60* and *Ktr88,* with estimated null allele frequencies for these loci between 0.12 (*Ktr58*) and 0.3 (*Ktr88*) (Additional file 2: Table S2-S3).

The five-locus dataset of 13 populations harboured 136 alleles across the five loci (range 21 to 38 per locus), while 147 alleles were detected across the eight loci in the Kosciuszko dataset (6–38 alleles/locus). Although observed heterozygosity was generally low (all H_O_ < 0.6), likely due to the presence of null alleles, genetic diversity measured by expected heterozygosity was high (mean *H*_*E*_ = 0.82) and varied little across sites (with the exception of the Baw Baw population, with *H*_*E*_ = 0.59) (Table 1).

**Table 1:** **Genetic variation in markers used for phylogeny reconstruction (** ***CO1*** **and** ***ITS1*** **) and population genetic analyses**

	***CO1***		***ITS1***	**Microsatellites (five shared loci)**			**Microsatellites (all typed loci)**		
**Location**	**Nucleotide diversity (** **π** **)**	**Number of haplotypes**	**Number of haplotypes**	***H***_***O***_	***H***_***E***_	***N***_***a***_	***H***_***O***_	***H***_***E***_	***N***_***a***_
*Kosciuszko region*									
Mt Jagungal	0.001	2	1	0.61	0.83	11.80	0.56	0.83	11.63
Mt Tate	0.002	4	1	0.51	0.81	12.40	0.51	0.82	11.88
Guthega	0.002	4	3	0.50	0.81	13.60	0.48	0.82	12.00
Mt Townsend	0.001	3	1						
Thredbo 1	0.001	2	1	0.56	0.82	12.40	0.53	0.79	10.25
Thredbo 2				0.51	0.83	13.20	0.48	0.80	11.25
Thredbo 3				0.57	0.81	13.80	0.50	0.77	11.25
*Victoria*									
Mt Bogong	0.008	2	1	0.46	0.88	13.40			
Mt Nelse	0.001	4	2						
Falls Creek	0.000	1	1	0.51	0.90	14.20			
Mt Cope	0.000	1	1	0.38	0.87	12.20			
Mt Jaithmathang	0.002	2	1						
Mt Hotham	0.041	5	2						
Mt Buffalo	0.002	5	1	0.26	0.80	9.80			
Mt Buller	0.001	2	1	0.49	0.78	8.80			
Mt Stirling	0.000	1	1	0.33	0.69	10.00			
Mt Baw Baw	0.002	3	5	0.27	0.59	7.40			

Genetic variation was measured as nucleotide diversity and haplotype number for *CO1*; nucleotide diversity was not calculated for *ITS1* as variation was very low. Observed heterozygosity (*Ho*), expected heterozygosity (*HE*) and mean allelic richness (*Na*) were calculated for five microsatellite loci that amplified across all populations, as well as for eight loci typed for the Kosciuszko populations.

### Population genetic structure

Deviations from HWE and the presence of null alleles means that estimates of F-statistics are problematic and should be treated cautiously. Nevertheless, we present *F*_*ST*_ values, corrected for null alleles, and *F’*_*ST*_ values as measures of divergence for comparability. The patterns of structure presented below, detected using F_ST_, principal components analysis and Bayesian clustering analyses are concordant.

There was significant pairwise differentiation between 73 of 78 site pairs in the five-locus dataset, and between 13 of 14 pairs in the Kosciuszko dataset, after a correction for null alleles using the ENA method (see Methods for details), although *F*_*ST*_ values were generally low (Additional file 3: Tables S4-S5). The exceptions were Falls Creek-Mt Cope (*F*_*ST*_ = 0.002, *F’*_*ST*_ = 0), Mt Buller-Mt Stirling (*F*_*ST*_ = 0.010, *F’*_*ST*_ = 0.112), Thredbo 2 – Mt Tate (*F*_*ST*_ = 0.014, F’_ST_ = 0.189), Thredbo 1-Thredbo 2 (*F*_*ST*_ = 0.002, *F’*_*ST*_ = 0.017) and Thredbo 2-Thredbo 3 (*F*_*ST*_ = 0.013, *F’*_*ST*_ = 0.057). The greatest differentiation was observed between populations from the Kosciuszko region and those from Victoria (mean *F*_*ST*_ = 0.100).

**Figure 4:**
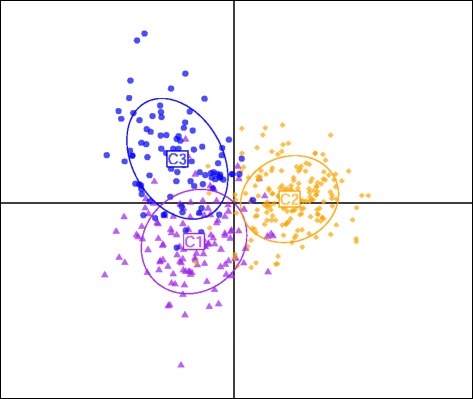
**DAPC plot for geographic clusters.** Results of a discriminant analysis of principal components, showing relationships among geographic population clusters. C1 = Bogong High Plains/Mt Buffalo, C2 = Kosciuszko region, C3 = Southern mountains (Mt Baw Baw, Mt Buller, Mt Stirling). Inertia ellipses are a graphical summary of points within each cluster, with the centroid representing the mean coordinates, width equal to the variances and slope equal to the covariance.

These broad-scale patterns were supported by the discriminant analysis of principal components, which indicated clear differentiation of geographic groups (Figure 4). In particular, the Kosciuszko region represented a robust cluster with 93% of individuals correctly assigned to the region, compared to 83% and 84% for the southern Victorian and Bogong High Plains clusters, respectively.

**Figure 5:**
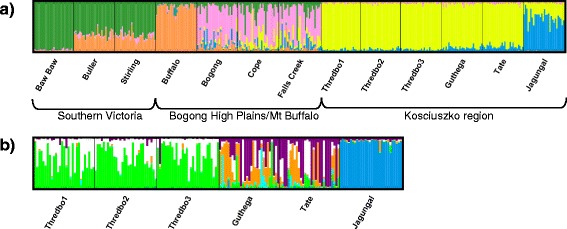
**TESS assignment analyses.** Posterior estimates of individual admixture proportions for **(a)** all populations (five-locus dataset) (*K*_*max*_ = 5) and **(b)** populations from the Kosciuszko region (*K*_*max*_ *= 6*). Admixture proportions represent the estimated proportion of an individual’s genome originating in each cluster and different clusters are represented by different colours.

Population structure was also assessed with the Bayesian assignment method implemented in TESS, which supported five clusters in the five-locus dataset (all populations) and six clusters in the Kosciuszko dataset (Figure 5). Strong regional divergence was again evident: three clusters were almost exclusively assigned to individuals from Victoria (<4% in the Kosciuszko region), while the remaining clusters were largely exclusive to the Kosciuszko region. The Bogong High Plains region showed equivalent admixture proportions from clusters associated with southern Victoria and the Kosciuszko region (10 – 18% admixture), in addition to a distinct local cluster representing an average 57% of individual admixture proportions. In Victoria, Mt Buffalo and Mt Baw Baw were characterised by very high single-cluster assignment (97% and 89% respectively). Similarly, in the Kosciuszko region, the isolated peak of Mt Jagungal showed high single-cluster assignment (average 93%).

### Spatial patterns of genetic divergence

We tested for isolation-by-distance (IBD) in both the microsatellite and *CO1* datasets, and examined this pattern in the Victorian and Kosciuszko regions alone, as well across all populations. There was significant isolation-by-distance in both the microsatellite (all populations (Five-locus): *r*^*2*^ = 0.640, p = 0.001; Victoria (Five-locus): *r*^*2*^ = 0.395, *p* = 0.020; Kosciuszko: *r*^*2*^ = 0.666, p = 0.001) and *CO1* (all populations: *r*^*2*^ = 0.213, *p* = 0.003; Victoria: *r*^*2*^ = 0.304, *p* = 0.005), datasets. Within the Kosciuszko region, however, *CO1* sequence divergence showed no relationship with geographic distance (*r*^*2*^ = 0.009, *p* = 0.367).

## Discussion

The topographic complexity of mountain landscapes, coupled with large-scale climatic fluctuations, has shaped the evolution and population structure of high-elevation species worldwide [2,7,10–12,27]. In the comparatively small (both in area and altitudinal range) and geologically stable Australian high country, we found genetic divergence associated with discontinuities in alpine and sub-alpine habitats. Four primary genetic groups were identified across the Kosciuszko region of New South Wales and mountain areas of Victoria, with the greatest structure evident in the latter. Here we discuss population structure within Kosciuszko and Victoria, genetic structure across major geographic breaks and the extent to which patterns in Australia parallel (and differ from) those in larger mountain systems.

### Within-region structure

The Kosciuszko alpine region contains Australia’s highest mountains and the most extensive area of alpine and sub-alpine habitat. Despite this, previous work in a variety of taxa [grasses: 18, skinks: 22, mammals: 23, insects: 25] has shown low genetic structure within this region. Likewise, we found no phylogeographic structure in *K. tristis*. An absence of isolation-by-distance suggests either very high gene flow over a long period of time or incomplete lineage sorting following relatively recent (re)colonisation [28,29]. We suggest the latter is more likely. The slopes on and near Mt Kosciuszko are the only areas of mainland Australia where glacial formations during the Pleistocene are known to have occurred [19]. In addition to concurrent changes in the distribution and structure of vegetation [30], *K. tristis* has poor cold tolerance (R Slatyer, unpublished data) and may thus have retreated to refugia during glacial periods. Subsequent recolonization during interglacial periods could lead to low diversity and low regional structure – a pattern which is seen among many alpine taxa in the Northern Hemisphere [27,31,32].

In contrast to the lack of phylogenetic structure, microsatellite data suggested significant population differentiation across small spatial scales (<20 km) coupled with a strong signature of isolation-by-distance. This could reflect more recent differentiation. In particular, we report a new genetic break, with unambiguous clustering of individuals from Mt Jagungal, which is separated from Kosciuszko proper by 25 km of sub-alpine plains at around ~ 1500 m elevation – also the lower-elevation limit of *K. tristis* in the area. Patterns of recent, fine-scale population differentiation (over distances < 50 km) are common across high-elevation taxa – even among species with high dispersal ability (e.g. [33–36]). Habitat features such as forest fragments within high-elevation meadows, topographic relief and water availability have been identified as putative dispersal barriers [33,37–39]. Although sub-alpine and alpine habitats are largely contiguous within the Kosciuszko region, as far north as Mt Jagungal, grasshoppers are patchily distributed throughout the landscape (R Slatyer & K Umbers, unpublished observations). This is likely to reflect microhabitat variation which, in turn, could be driving the genetic differentiation across small geographic distances within the Kosciuszko region.

Victoria’s high country is highly fragmented, with disjunct mountain regions separated by 50 to 100 km. In line with our predictions that populations from this region would thus show stronger genetic structure than those from Kosciuszko, divergence and diversity were both higher within Victoria. Despite this pattern of generally high regional divergence, however, the Bogong High Plains and Mt Buffalo showed low differentiation. Mt Buffalo contains several endemic flora and fauna species [40,41], showed strong phylogeographic isolation in four other alpine invertebrates [17] and emerged as one of few distinct genetic clusters in wind-dispersed alpine *Poa* grasses [18]. It was therefore surprising to find no support for a distinct Mt Buffalo clade in our phylogenetic analyses, particularly as this would correspond to the subspecies described from morphological data [24]. As for populations within the Kosciuszko region, however, assignment analyses based on microsatellite data suggest that this population is genetically distinct from those of the adjacent Bogong High Plains. Mutations arise rapidly in microsatellites [42], and this differentiation could suggest recent population isolation.

### Evolutionary history of the Australian alps

Rapid cooling and drying in southeastern Australia during the late Miocene to Pliocene (5–2.5 Ma) is thought to have promoted the evolution of the cold-adapted sub-alpine and alpine biota, with this species assemblage persisting through subsequent climatic shifts [43–45]. This corresponds to estimated dates of divergence among the four mainland *Kosciuscola* species [25]. Divergence time estimates for the southern Victorian (0.5 – 1.8 Ma), and subsequently, Mt Baw Baw and Mt Buller clades (0.1 – 0.8 Ma), are consistent with the intense climate cycling of the Pleistocene [20,46,47]. A lack of current gene flow is indicated by an absence of widespread haplotypes and haplotype sharing only between proximate populations [10].

Depression of the snowline during glacial periods is thought to have resulted in continuous alpine/sub-alpine conditions from Mt Jagungal into southern Victoria [15,16]. While this depression might have facilitated range expansion and, consequently, low differentiation across the alpine area as a whole [9,48], previous studies on geographic subsets of Australia’s high country have found high endemism among mountain suggesting relatively stable population histories and isolation among mountains [17,21,22]. In particular, considering both the sequence and microsatellite datasets, we identify four primary genetic groups: two southern Victorian groups comprising Mt Baw Baw and Mt Buller/Mt Stirling; a Bogong High Plains cluster comprising the Bogong High Plains proper and Mt Buffalo; and a Kosciuszko cluster.

One of the most surprising results to emerge in this study was the inconsistent support for the (relatively) well-studied phylogeographic break between the Kosciuszko and Bogong High Plains regions, across the Murray River valley. Approximately 100 km separates alpine/sub-alpine habitats on either side of the valley but two of five previous studies [18,25] have not found significant genetic structure. From both sequences and microsatellite data, we suggest that there is significant divergence between *K. tristis* populations from Mt Kosciuszko and the Bogong High Plains. Perhaps more importantly, however, our data suggest that the Murray River valley (which also forms a State border) may not be the greatest point of divergence among alpine taxa (see also [18]).

### The Australian alps in a global perspective

For high-elevation taxa in North America and Europe, phylogeographic patterns reflect contrasting population histories of glacial and interglacial expansion and contraction (e.g. [2,4,11,48,49]). In many respects, our phylogenetic results mirror those from mountains in the Northern hemisphere – phylogeographic breaks among widespread mountain regions contrast with shallow historical divergence within ranges [6,11,49]. However, mountain regions in Australia are of a different scale to those most often studied – peaks are separated by tens of kilometres, rather than by hundreds of kilometres, and alpine/sub-alpine habitat is confined to just 700 vertical metres. Further, all current high-mountain regions on mainland Australia are thought to have been connected by alpine/sub-alpine conditions during glacial periods. Given that the processes typically invoked to explain distribution shifts and demographic change - such as ice sheet formation – are not applicable to much of the Australian high country, and that distances among mountain regions are relatively small, it is remarkable that strong lineage divergence (such as for the southern Victorian mountains) is clearly evident. Indeed, sequence divergence was akin to estimates of inter-specific divergence among alpine grasshoppers in the North American Rocky Mountains [7], and across the mountains of Europe [50].

## Conclusions

Despite the small spatial scale and past intermittent connectivity of Australian alpine and sub-alpine habitats, consistent patterns of divergence among the two molecular datasets indicate differentiation, associated with geographic breaks in alpine/sub-alpine habitat, that has persisted through glacial cycles and into the present. Populations from peaks within the highly fragmented Victorian high country showed much greater population structure than those from the more continuous Kosciuszko region and structure patterns indicate that the former is comprised of at least three distinct genetic groups. The microsatellite data, although problematic, suggests more fine-scale and possibly more recent structure than either *CO1* or *ITS1,* and genetically distinct populations corresponding to the northern- and southern-most parts of the species’ range and to the described sub-species *K. tristis restrictus*. The evolutionary histories of these peaks, particularly the under-studied Mt Jagungal, need to be assessed further. The wide alpine/sub-alpine distribution and ecological characteristics of *K. tristis* make it a good model for exploring associations between genetic markers and adaptive processes across the Australian high country.

## Methods

### Study species and sampling

*Kosciuscola tristis* Sjösted (1933) (Orthoptera: Acrididae) is a small (15–30 mm) grasshopper, endemic to Australia’s alpine region [51]. The species has a patchy distribution across small geographic scales, but population density can be very high during the peak adult activity period between February and April. The species is univoltine with discrete generations, all adults dying by late-May and eggs hatching in early summer. Of the four grasshopper species endemic to the sub-alpine and alpine regions of the Australian mainland, *K. tristis* has the narrowest altitudinal range. It is found between 1500 m and 2200 m with a known distribution spanning five distinct mountain areas across two States: New South Wales (Kosciuszko alpine region) and Victoria (Bogong High Plains, Mt Buffalo plateau, Mt Buller/Mt Stirling and the Baw Baw plateau) (Figure 1).

Between January and May of 2012 and 2013, a total of 396 adult *K. tristis* were collected by hand from 15 mountains spanning *K. tristis’* full known geographic distribution (Figure 1, Additional file 1: Table S1). Some mountains were separated by low-elevation, while others represent peaks within continuous alpine/sub-alpine habitat. The geographic distance between sampled locations ranged from 1.7 km to 284 km, but adjacent mountains were always less than 100 km apart. One hind leg was removed from each individual in the field and stored in 100% ethanol until DNA extraction.

### Phylogeny reconstruction: DNA extraction, amplification and sequencing

Total genomic DNA was extracted from muscle tissue from the femur. Tissue was placed in a microcentrifuge tube and crushed with a glass bead and mixer mill at 20,000 Hz for 2 min. Samples were centrifuged (30 sec at 13,000 rpm) after which 150 μL of 5% Chelex-100 resin® (BioRad, Hercules, USA) and 3 μL proteinase K (10mg/mL) (Roche, Basel, Switzerland) were added. Samples were incubated at 56°C for 3 hours, followed by 95°C for 10 min [52].

Phylogenetic analyses were performed using DNA sequence data from fragments of the mitochondrial cytochrome oxidase subunit I (*CO1*) gene and the nuclear internal transcribed spacer 1 (*ITS1*) region for five individuals per collection site (total 75 individuals). Polymerase Chain Reactions (PCRs) were used to amplify a 801 base-pair (bp) fragment of the *CO1* gene using primer pairs C1-J-2183 and TL2-N-3014 [53], and a 573 bp fragment of the *ITS1* gene using primer pairs CAS18sF1 and CAS5p8sB1d [54]. PCR was carried out under the following reaction conditions: *CO1*: 94°C for 4 min, 35 cycles of 94°C for 30 sec, 45°C for 30 sec, 72°C for 30 sec, then 72°C for 5 min; *ITS1*: 94°C for 4 min, 35 cycles of 94°C for 30 sec, 64°C for 40 sec, 72°C for 30 sec, then 72°C for 5 min. PCRs were performed in 25 μL (see Additional file 4: Table S6 for reaction concentrations) and products were sequenced on an ABI 3730 DNA analyser (Macrogen Inc, Korea). *Kosciuscola cognatus,* a congener occupying the sub-alpine zone, was sequenced as an outgroup taxon, using identical PCR conditions.

### Phylogeny reconstruction: sequence analysis and divergence time estimation

DNA sequences were aligned using muscle [55] with default settings and refined manually in geneious 6.1.7 [56]. *CO1* sequences were translated into amino acid sequences and checked for internal stop codons to determine product authenticity and the correct reading frame. Unique haplotypes in each dataset were identified and used for subsequent phylogenetic analyses [Genbank: KJ870103-KJ870137 & KJ870139-KJ870149] (Additional file 5: Table S7). We used two approaches to test phylogeographic structure.

First, we used maximum likelihood (ML) to estimate phylogenetic relationships of *CO1* haplotypes. The data were partitioned by codon position and RAxML 7.4.2 [57] was used to estimate a maximum likelihood tree using the gtrgamma model and a rapid bootstrapping analysis [58] with 1000 iterations. RAxML was implemented in raxmlGUI 1.3 [59]. Two sequences from the congener *K. cognatus* were used to root the tree [Genbank: KJ870138, KM407143].

Second, as there was low variation in the *ITS1* sequences (12 haplotypes), we generated a phylogenetic network using a median-joining approach [60] implemented in network 4.6.1.1 [58,61], with standard settings. *Kosciuscola cognatus* was again included as an outgroup [Genbank: KJ870150].

We used the mitochondrial *CO1* sequences to estimate divergence time among clades. In particular, we were interested in the timing of divergence among Victorian and Kosciuszko populations. No fossil of geological evidence was available for node calibration. Instead, we estimated divergence times using two divergence rates: 3.5% Myr^−1^, based on a recent insect molecular clock estimate [62] and 2.3% Myr^−1^, which has been used for grasshoppers in the past [7]. Although it is unrealistic to assume a fixed substitution rate across taxa and lineages [63], we proceeded with this method to allow comparisons with other studies and to place population divergence in a rough time period. Sequence divergence was measured as uncorrected p-distance, calculated with the *ape* package [64] in R 3.1.0 [65].

Finally, we calculated average nucleotide diversity (π) for each collection site using DnaSP v5.10.1 [66], and mean p-distance between haplotypes in Victoria and in the Kosciuszko region, to compare levels of diversity among these areas. Diversity statistics were calculated only for the *CO1* data, as *ITS1* sequences showed extremely low diversity.

### Population genetic structure: DNA extraction, amplification and genotyping

Genomic DNA was extracted from femur tissue, which was placed in a 96-well plate with 3 μL proteinase K and 150 μL of 5% Chelex. The plate was then incubated at 56°C for 16 h [67]. Population genetic analyses were performed using 29–31 individuals from 13 collection sites (Table 1), three of which were from a single elevation gradient (at elevations of 1901, 1781, and 1681 m a.s.l) near Thredbo, NSW (Thredbo 1, 2, and 3 respectively). Eight microsatellite loci were amplified across two multiplexes, using primers developed by Umbers*, et al.* [68] for *K. tristis* (Multiplex 1: loci *Ktr29*, *Ktr73*, *Ktr76* and *Ktr82*; Multiplex 2: loci *Ktr30*, *Ktr58*, *Ktr60* and *Ktr88*). Microsatellites were designed and tested with *K. tristis* from the Thredbo 2 population and details of the design methodology are given in [68]. PCRs were performed in 11 μL volumes containing 5 μL QIAGEN Multiplex PCR Master Mix (QIAGEN Inc., Valencia, CA, U.S.A), 0.1 μM forward primer, 0.2 μM reverse primer, 0.1 μM fluorescent tags (FAM, VIC, NED, PET), and approximately 7 ng of genomic DNA [69]. PCR cycling was performed under the following conditions: 95°C for 15 minutes, 40 cycles of 94°C for 30 s, 59°C for 90 s, and 72°C for 60 s; followed by 30 min at 60°C. PCR products were size-separated on an AB3730 DNA analyser, standardised against a GeneScan™ Liz®500 size standard (Life Technologies, CA, USA) at the Australian Genome Research Facility, Melbourne. Fragment sizes were scored manually using Geneious v6.1.7 [56]. The loci *Ktr73, Ktr82* and *Ktr60* had high failure rates (>60 %) for all Victorian populations. Subsequent analyses were therefore run with two datasets (unless indicated otherwise): (1) all populations with the five remaining loci (five-locus dataset), and (2) the six populations from the Kosciuszko region (Kosciuszko dataset).

### Population genetic structure: diversity and differentiation

Prior to analysis, individual microsatellite loci were checked for linkage disequilibrium, departure from Hardy-Weinberg equilibrium (HWE) and null alleles. The data were initially screened for anomalies using microchecker [26]. Genepop 4.2.2 [70] was used to test for linkage disequilibrium (LD) among loci in each population, using default parameters, and to estimate deviations from HWE. Significance values were adjusted for multiple comparisons with False Discovery Rates [71], implemented in R 3.1.0 [65]. The frequency of null alleles at each locus was estimated using FreeNA [72] and diversity statistics, including allelic richness and estimates of observed (*H*_*O*_*)* and expected (*H*_*E*_) heterozygosity, were calculated with GenAlEx 6.5b3 [73,74].

We used three methods to examine population structure. First, we used FreeNA to calculate global and pairwise *F*_*ST*_ values, correcting for the presence of null alleles with the ENA method [72]. We also computed the standardised statistic *F’*_*ST*_ in Genodive 2.0b23 [75]. *F*_*ST*_ values are influenced by the amount of genetic variation within populations, with the maximum attainable value of *F*_*ST*_ decreasing as heterozygosity increases [76]. Thus, differentiation among populations is often underestimated, particularly for highly variable molecular markers such as microsatellites, and several authors have recommended calculation of standardised statistics, such as *F’*_*ST*_*,* as a more appropriate measure of genetic differentiation [77–79]. Both statistics were calculated for consistency and comparability with other studies.

Second, we used a discriminant analysis of principal components (DAPC; [80]) to investigate the relationship among geographic regions. This approach does not have underlying assumptions of HWE and was thus appropriate for our dataset (see Results). The method attempts to optimise between-cluster variation whilst minimising variation within clusters, thus producing a clearer distinction of clusters [80]. As populations were spatially discrete, DAPC was run using *a priori* clusters [80]: (a) the southern Victorian mountains (Mt Baw Baw, Mt Buller, Mt Stirling), (b) the Bogong High Plains and Mt Buffalo, and (c) Kosciuszko populations. DAPC was run in the *adegenet* package (v1.4-1) [80] in R, with the first 50 principal components retained (capturing 80% of variation), along with all the discriminant functions. As we were interested in genetic relationships among regions, this analysis was only run using the five-locus dataset.

Third, we examined population structure within the Kosciuszko region and across the study area using a Bayesian clustering approach, implemented in tess 2.3 [81,82]. TESS incorporates individual geographic coordinates as *a priori* information and can thus incorporate spatial trends and spatial autocorrelation in estimates of individual ancestry [82]. As tess only accepts individual coordinates, population coordinates were permuted with a standard deviation of 0.005°. We used the conditional autoregressive (CAR) admixture model, with a burn-in of 50,000 steps followed by 100,000 MCMC steps. The admixture parameter (α) and interaction parameter (ρ) were initially set to α = 1 and ρ *= 0.6, then automatically updated. We searched for the optimal number of clusters (K) using 10 runs at K = 2 to K = 10.* The most likely number of clusters was selected from the lowest Deviance Information Criterion (DIC), averaged over these 10 runs. We then did 100 additional runs at the optimal value of *K* and the 20 runs with the lowest DIC were averaged using clumpp 1.1.2 [83] with the ‘greedy’ algorithm and default settings. The level of admixture in each population was displayed graphically with distruct 1.1 [84].

### Spatial patterns of genetic divergence

We examined spatial patterns of divergence using both the *CO1* and microsatellite data. First, we tested for a relationship between genetic distance (p-distances for *CO1* data and *F’*_*ST*_ for microsatellites) and log-transformed geographic distance (isolation-by-distance) [85]. For both mtDNA and microsatellites, we tested for IBD across all populations, populations from Victorian only, and populations from NSW only. Mantel tests were run using the *vegan* package [86] in R, with 999 permutations to test statistical significance.

## Availability of supporting data

The data sets supporting the results of this article are available in the figshare repository [http://dx.doi.org/10.6084/m9.figshare.1165613] [87], or are contained within the article and its additional files.

## Additional files

Additional file 1: Table S1. Sampling locations and sample sizes for *CO1, ITS1* and microsatellite analyses. Additional file 2: Table S2-S3. Population- and locus-specific null allele frequencies. Additional file 3: Tables S4-S5. Pairwise *F*_*ST*_ and *F’*_*ST*_ matrices for microsatellite data. Additional file 4: Table S6. Details of reagent concentrations for polymerase chain reactions to amplify CO1 and ITS1 sequences. Additional file 5: Table S7. Genbank accession numbers and haplotype assignment.
