# Supplementary material for: Strong genetic structure corresponds to small-scale geographic breaks in the Australian alpine grasshopper Kosciuscola tristis
Source: BMC Evol Biol. 2014 Oct 2;14:204. doi: 10.1186/s12862-014-0204-1 (PMC4203917; doi:10.1186/s12862-014-0204-1)
Supplement: Additional file 1: Table S1. — Sampling locations and sample sizes for CO1, ITS1 and microsatellite analyses. [file 12862_2014_204_MOESM1_ESM.pdf]

Additional file 1.

Table 1S. Sampling locations and sample sizes for *CO1, ITS1* and microsatellite analyses.

**Location**	**Latitude**	**Longitude**	**Elevation (m a.s.l)**	***CO1/ITS1* sample size**	**Microsatellites sample size**
Kosciuszko region					
Mt Jagungal	-36.1481°	148.3875°E	2054	5	31
Mt Tate	-36.3726°	148.3652°E	1671	5	29
Guthega	-36.3843°	148.3764°E	1698	5	30
Mt Townsend	-36.4225°	148.2588°E	2205	5	0
Thredbo 1	-36.5015°	148.2728°E	1901	5	29
Thredbo 2	-36.5093°	148.2694°E	1781	0	30
Thredbo 3	-36.5173°	148.2647°E	1681	0	30
Victoria					
Mt Bogong	-36.7559°	147.3266°E	1791	5	30
Mt Nelse	-36.8434°	147.3445°E	1805	5	0
Falls Creek	-36.8700°	147.2777°E	1744	5	30
Mt Cope	-36.9272°	147.2812°E	1781	5	29
Mt Jaithmathang	-36.8903°	147.1923°E	1844	5	0
Mt Hotham	-36.9890°	147.1470°E	1782	5	0
Mt Buffalo	-36.7553°	146.7914°E	1536	5	29
Mt Buller	-37.1455°	146.4308°E	1774	5	30
Mt Stirling	-37.1216°	146.4989°E	1627	5	30
Mt Baw Baw	-37.8335°	146.3081°E	1476	5	29
