# Supplementary material for: Strong genetic structure corresponds to small-scale geographic breaks in the Australian alpine grasshopper Kosciuscola tristis
Source: BMC Evol Biol. 2014 Oct 2;14:204. doi: 10.1186/s12862-014-0204-1 (PMC4203917; doi:10.1186/s12862-014-0204-1)
Supplement: Additional file 2: Table S2-S3. — Population- and locus-specific null allele frequencies. [file 12862_2014_204_MOESM2_ESM.pdf]

Additional file 2. Population- and locus-specific allele frequencies

Table 2S. Average population-specific null allele frequencies

**Population**	**Null allele frequency**	
	**mean**	**SD**
Five-locus dataset		
Thredbo 1	0.12	0.08
Thredbo 2	0.11	0.10
Thredbo 3	0.09	0.09
Guthega	0.12	0.11
Jagungal	0.10	0.12
Tate	0.12	0.11
Buffalo	0.21	0.09
Bogong	0.20	0.13
Cope	0.20	0.10
Falls Creek	0.19	0.07
Buller	0.19	0.10
Stirling	0.18	0.09
Baw Baw	0.19	0.14
Kosciuszko dataset		
Thredbo 1	0.16	0.07
Thredbo 2	0.20	0.07
Thredbo 3	0.17	0.12
Guthega	0.21	0.12
Tate	0.19	0.12
Jagungal	0.20	0.11

Table 3S. Average locus-specific null allele frequencies.

**Locus**	**Null allele frequency**	
	**mean**	**SD**
Five-locus dataset		
Ktr29	0.09	0.03
Ktr76	0.04	0.05
Ktr73	0.18	0.04

Ktr82	0.18	0.08
Ktr58	0.20	0.08
Ktr88	0.30	0.03
Ktr30	0.25	0.06
Ktr60	0.30	0.05
Kosciuszko dataset		
Ktr29	0.05	0.04
Ktr76	0.12	0.10
Ktr58	0.14	0.080
Ktr88	0.30	0.05
Ktr30	0.17	0.07
