# Supplementary material for: Strong genetic structure corresponds to small-scale geographic breaks in the Australian alpine grasshopper Kosciuscola tristis
Source: BMC Evol Biol. 2014 Oct 2;14:204. doi: 10.1186/s12862-014-0204-1 (PMC4203917; doi:10.1186/s12862-014-0204-1)
Supplement: Additional file 3: Tables S4-S5. — Pairwise F ST and F’ ST matrices for microsatellite data. [file 12862_2014_204_MOESM3_ESM.pdf]

Additional file 3. Pairwise F_{ST} and F'_{ST} matrices for microsatellite data.

Table 4S. Pairwise F_{ST} (below diagonal) and F'_{ST} (above diagonal) matrices for the 5-locus dataset. F_{ST} values were corrected for null alleles with the ENA method (Chapuis & Estoup 2007). Stastical significance was tested for F_{ST} values, using 1000 bootstrap iterations and corrected for multiple comparisons using the False Discovery Rates method. All pairwise differences are statistically significant ($p < 0.05$) except those in bold.

	**Buffalo**	**Bogong**	**Cope**	**Falls**	**Baw Baw**	**Buller**	**Stirling**	**Guthega**	**Jagungal**	**Tate**	**Thredbo 1**	**Thredbo 2**	**Thredbo 3**
**Buffalo**		0.535	0.484	0.497	0.408	0.38	0.568	0.456	0.561	0.469	0.524	0.495	0.515
**Bogong**	0.079		0.172	0.227	0.456	0.432	0.284	0.643	0.623	0.556	0.514	0.485	0.636
**Cope**	0.093	0.015		0	0.295	0.309	0.355	0.402	0.540	0.344	0.388	0.364	0.433
**Falls**	0.083	0.013	**0.002**		0.424	0.313	0.327	0.422	0.401	0.384	0.397	0.321	0.434
**Baw Baw**	0.108	0.050	0.057	0.056		0.207	0.249	0.357	0.485	0.34	0.508	0.419	0.503
**Buller**	0.070	0.044	0.039	0.035	0.047		0.112	0.571	0.505	0.516	0.585	0.527	0.555
**Stirling**	0.077	0.036	0.043	0.034	0.055	**0.010**		0.657	0.559	0.573	0.644	0.549	0.672
**Guthega**	0.156	0.083	0.057	0.057	0.118	0.102	0.109		0.414	0.028	0.112	0.095	0.074
**Jagungal**	0.148	0.080	0.066	0.056	0.111	0.093	0.096	0.059		0.409	0.463	0.376	0.427
**Tate**	0.158	0.081	0.056	0.059	0.108	0.099	0.100	0.010	0.064		0.189	0.117	0.15
**Thredbo 1**	0.160	0.079	0.062	0.060	0.127	0.106	0.110	0.013	0.068	0.027		0.023	0.085
**Thredbo 2**	0.149	0.067	0.049	0.047	0.105	0.089	0.092	0.015	0.055	**0.014**	0.005		0.022
**Thredbo 3**	0.172	0.096	0.068	0.070	0.137	0.112	0.121	0.007	0.069	0.021	0.012	0.008	

Table 5S. Pairwise F_{ST} (below diagonal) and F'_{ST} (above diagonal) matrices for the Kosciuszko dataset. F_{ST} values were corrected for null alleles with the ENA method (Chapuis & Estoup 2007). Stastical significance was tested for F_{ST} values, using 1000 bootstrap iterations and corrected for multiple comparisons using the False Discovery Rates method. All pairwise differences are statistically significant ($p < 0.05$) except those in bold.

	**Guthega**	**Jagungal**	**Tate**	**Thredbo 1**	**Thredbo 2**	**Thredbo 3**
**Guthega**		0.312	0.048	0.162	0.123	0.144
**Jagungal**	0.048		0.307	0.326	0.291	0.298
**Tate**	0.011	0.044		0.166	0.079	0.181
**Thredbo 1**	0.029	0.065	0.031		0.017	0.106
**Thredbo 2**	0.023	0.048	0.013	**0.002**		0.057
**Thredbo 3**	0.023	0.055	0.028	0.018	**0.013**	
