# Supplementary material for: Strong genetic structure corresponds to small-scale geographic breaks in the Australian alpine grasshopper Kosciuscola tristis
Source: BMC Evol Biol. 2014 Oct 2;14:204. doi: 10.1186/s12862-014-0204-1 (PMC4203917; doi:10.1186/s12862-014-0204-1)
Supplement: Additional file 4: Table S6. — Details of reagent concentrations for polymerase chain reactions to amplify CO1 and ITS1 sequences. [file 12862_2014_204_MOESM4_ESM.pdf]

Additional file 4.

Table 6S. Details of reagent concentrations for polymerase chain reactions to amplify *CO1* and *ITS1* sequences.

**Reagent**	**PCR concentration**	**Volume (ul) in 25 µL reaction**
CO1		
BSA	0.5 µg/µL	1.25
MgCl2	0.75 mM	0.75
10x Thermo pro buffer	1 x	2.5
dNTPs	200 µM	5
Forward primer	0.016 µM	1
Reverse primer	0.016 µM	1
NEB taq	1.25 u	0.25
DNA	~0.7 ng/µL	5
ITS1		
BSA	0.5 µg/µL	1.25
MgCl2	0.75 mM	0.75
10x Thermo pro buffer	1 x	2.5
dNTPs	200 µM	5
Forward primer	0.016 µM	1
Reverse primer	0.016 µM	1
NEB taq	1.25 u	0.25
DNA	~0.7 ng/µL	5
