# Supplementary material for: Strong genetic structure corresponds to small-scale geographic breaks in the Australian alpine grasshopper Kosciuscola tristis
Source: BMC Evol Biol. 2014 Oct 2;14:204. doi: 10.1186/s12862-014-0204-1 (PMC4203917; doi:10.1186/s12862-014-0204-1)
Supplement: Additional file 5: Table S7. — Genbank accession numbers and haplotype assignment. [file 12862_2014_204_MOESM5_ESM.pdf]

Additional file 5. Genbank accession numbers and haplotype assignment

Table 7S. Genbank accession numbers for *Kosciuscola tristis* sequences used to construct the *CO1* and *ITS1* phylogenies. The collection sites represented by each haplotype, and number of individuals, are also provided.

**Haplotype number**	**Genbank accession number**	**Collection site (*N* individuals)**
CO1		
1	KJ870103	Mt Bogong (4)<br>Mt Jaithmathang (4)<br>Mt Cope (5)<br>Falls Creek (5)
2	KJ870104	Mt Buller (4)
3	KJ870105	Thredbo (4)
4	KJ870106	Mt Jaithmathang (1)
5	KJ870107	Mt Hotham (2)
6	KJ870108	Mt Buller (1)
7	KJ870109	Mt Stirling (5)
8	KJ870110	Mt Baw Baw (1)
9	KJ870111	Mt Baw Baw (1)
10	KJ870112	Mt Baw Baw (3)
11	KJ870113	Mt Tate (1)
12	KJ870114	Mt Jagungal (4)
13	KJ870115	Mt Tate (1)<br>Guthega (2)
14	KJ870116	Thredbo (1)
15	KJ870117	Mt Tate (1)<br>Guthega (1)
16	KJ870118	Mt Tate (2)
17	KJ870119	Mt Nelse (1)
18	KJ870120	Mt Nelse (2)
19	KJ870121	Mt Bogong (1)
20	KJ870122	Mt Jagungal (1)
21	KJ870123	Mt Buffalo (1)
22	KJ870124	Mt Buffalo (1)
23	KJ870125	Mt Buffalo (1)
24	KJ870126	Mt Buffalo (1)

25	KJ870127	Mt Buffalo (1)
26	KJ870128	Mt Nelse (1)
27	KJ870129	Mt Nelse (1)
28	KJ870130	Mt Townsend (2)
29	KJ870131	Mt Townsend (2)
30	KJ870132	Mt Townsend (1)
31	KJ870133	Guthega (1)
32	KJ870134	Guthega (1)
33	KJ870135	Mt Hotham (1)
34	KJ870136	Mt Hotham (1)
39	KJ870137	Mt Hotham (1)
ITS1		
1	KJ870139	Mt Baw Baw (1)
2	KJ870140	Mt Baw Baw (1)
3	KJ870141	Mt Baw Baw (1)
4	KJ870142	Mt Baw Baw (1)
5	KJ870143	Mt Baw Baw (1)
6	KJ870144	Mt Bogong (4)<br>Mt Buffalo (5)<br>Mt Cope (5)<br>Falls Creek (5)<br>Mt Jaithmathang (5)<br>Mt Nelse (4)
7	KJ870145	Mt Buller (5)<br>Mt Hotham (4)<br>Mt Stirling (5)
8	KJ870146	Guthega (1)
9	KJ870147	Guthega (4)<br>Mt Jagungal (5)<br>Mt Tate (5)<br>Thredbo (5)<br>Mt Townsend (5)
10	KJ870148	Mt Nelse
11	KJ870149	Mt Hotham
